# Recovery of Glucose from Residual Starch of Sago Hampas for Bioethanol Production

**DOI:** 10.1155/2013/935852

**Published:** 2012-12-27

**Authors:** D. S. Awg-Adeni, K. B. Bujang, M. A. Hassan, S. Abd-Aziz

**Affiliations:** ^1^Department of Bioprocess Technology, Faculty of Biotechnology and Biomolecular Sciences, Universiti Putra Malaysia, 43400 Serdang, Malaysia; ^2^Department of Molecular Biology, Faculty of Resource Sciences and Technology, Universiti Malaysia Sarawak, 94300 Kota Samarahan, Malaysia

## Abstract

Lower concentration of glucose was often obtained from enzymatic hydrolysis process of agricultural residue due to complexity of the biomass structure and properties. High substrate load feed into the hydrolysis system might solve this problem but has several other drawbacks such as low rate of reaction. In the present study, we have attempted to enhance glucose recovery from agricultural waste, namely, “sago hampas,” through three cycles of enzymatic hydrolysis process. The substrate load at 7% (w/v) was seen to be suitable for the hydrolysis process with respect to the gelatinization reaction as well as sufficient mixture of the suspension for saccharification process. However, this study was focused on hydrolyzing starch of sago hampas, and thus to enhance concentration of glucose from 7% substrate load would be impossible. Thus, an alternative method termed as cycles I, II, and III which involved reusing the hydrolysate for subsequent enzymatic hydrolysis process was introduced. Greater improvement of glucose concentration (138.45 g/L) and better conversion yield (52.72%) were achieved with the completion of three cycles of hydrolysis. In comparison, cycle I and cycle II had glucose concentration of 27.79 g/L and 73.00 g/L, respectively. The glucose obtained was subsequently tested as substrate for bioethanol production using commercial baker's yeast. The fermentation process produced 40.30 g/L of ethanol after 16 h, which was equivalent to 93.29% of theoretical yield based on total glucose existing in fermentation media.

## 1. Introduction

In recent years, there has been an increasing trend towards more efficient utilization of agro-industrial by-products for conversion to a range of value-added bioproducts, including biofuels, biochemicals, and biomaterials [1]. As an initiative, this study was formulated to utilize sago hampas as an alternative substrate for glucose production, which will be used as feedstock for bioethanol production. Sago hampas is a starchy lignocellulosic by-product generated from pith of *Metroxylon sagu* (sago palm) after starch extraction process [2]. *Metroxylon sagu* Rottb. is an increasingly important socioeconomic crop in Southeast Asia whereas New Guinea is believed to be its center of diversity [3]. In Malaysia, the state of Sarawak is recognized as the largest sago-growing areas, which is currently the world's biggest exporter of sago starch, exporting annually about 44,000 t of starch mainly to Peninsular Malaysia, Japan, Singapore, and other countries [4]. The isolation of sago starch involves debarking, rasping, sieving, settling washing, and drying [2]. However, the mechanical process currently employed to extract sago starch is inefficient and often fails to dislodge residual starch embedded in the fibrous portion of the trunks [3]. On dry basis, sago hampas contains 58% starch, 23% cellulose, 9.2% hemicellulose, and 4% lignin [5]. Approximately, 7 t of sago hampas is produced daily from a single sago starch processing mill [6]. Currently, these residues which are mixed together with wastewater are either washed off into nearby streams or deposited in the factory's compound. These circumstances, in time, may potentially lead to serious environmental problems.

Several studies on the utilization of sago hampas as animal feed, compost for mushroom culture, for hydrolysis to confectioners' syrup, particleboard manufacture, and as substrate for local microbes to produce reducing sugars and enzyme have been described elsewhere [7–10]. The study on extracting starch from sago hampas has been carried out by Manan et al. [11] using 2 types of commercial cell wall degrading enzymes, Pectinex Ultra SP-L and Ultrazyme 100G, and they extracted up to 42% more starch from residue with a wider granule size distribution than the untreated residue. However, this study was focused on extracting starch without any continuation on glucose production by enzymatic hydrolysis process. Other related studies were mainly focusing on the ability of local isolate enzymes to degrade all components of sago hampas into reducing sugars which, however, shows low productivity of sugars production [10].

Starch processing is a technology utilizing enzymatic liquefaction and saccharification, which produces a relatively clean glucose stream that is fermented to ethanol by *Saccharomyces *yeasts [12]. The supplement of glucoamylase with debranching enzyme, pullulanase which hydrolyze *α*-1,6 links in the chain to obtain glucose from gelatinized starch, is practically useful, since both enzymes have the same range of optimum pH [13]. Glucose from sago starch is used as substrate in the fermentation industry and for the production of high-fructose syrup [14]. Ethanol is gaining importance as a fuel additive, or as a conventional non-renewable fuel replacement [15]. The substrate is the main cost component for industrial ethanol production, and it is essential that ethanol production should be carried out with cheap substrate such as starch or cellulose [16]. In a study on simultaneous saccharification and fermentation of ethanol from sago starch with coimmobilized amyloglucosidase and *Zymomonas mobilis* MTCC 92 by submerged fermentation, a maximum ethanol concentration of 55.3 g/L was obtained using a starch concentration of 150 g/L [17]. However in this study, trapped starch in sago hampas was used as substrate for bioethanol fermentation.

In most ethanol fermentation, the greater substrate load would lead to increased ethanol concentration and, therefore, improve the efficiency of downstream processing. Moreover, the ability to work at high-solid concentrations is an important parameter in enzymatic hydrolysis process as it will influence the energy balance and economic viability of bioethanol production [18]. However, in real scenario, lower concentration of reducing sugars was often obtained from hydrolysis process of agricultural residue due to the complexity of the biomass structure and properties. Thus, water evaporation or ultrafiltration is part of the technique applied to get high sugars concentration from the hydrolysate, which in turn affects overall costs and processing time.

In this paper, we present the method for obtaining high glucose concentration from waste starch of sago hampas via three-cycle enzymatic hydrolysis process. Subsequently the glucose in the hydrolysate will be tested to determine their fermentability for bioethanol production using commercial bakery yeast.

## 2. Materials and Methods

### 2.1. Sago Hampas

Sago hampas was obtained from Herdsen Sago Mill in Sarawak, Malaysia. The hampas was packed into porous plastic bags and left to stand for 1-2 days. This was done to allow water from the wet hampas to drain off naturally. Prior to composition analysis, the sago hampas was oven dried at 65°C for 24 hours before grounded to pass a 1 mm screen. Dried samples were then analyzed for moisture content in order to quantify the suitable amount of buffer to be added for enzymatic hydrolysis process [19].

### 2.2. Enzymes

The commercial saccharification enzyme used in this study was dextrozyme (5.56 U/mL), provided by NOVOZYME, Denmark. This enzyme was a mixture of glucoamylase from *Aspergillus niger* and pullulanase from *Bacillus acidopullulyticus*. All other reagents used for this study were of analytical grade.

### 2.3. Saccharification of Starch in Sago Hampas

A suspension of sago hampas, 5% (w/v) was prepared in 0.1 M KH_2_PO_4_ buffer solution at pH 4. The suspension was boiled for 15 min for gelatinization process and subsequently cooled down to 60°C. A 0.3% (v/w) of Dextrozyme enzyme (Novozyme, Denmark) was then added into the mixture. A stirrer (Stuart SS30) was used for mixing the suspension to ensure homogeneity between enzyme and substrate. The suspension was left submerged in a water bath at 60°C for 60 min. The flask of suspension was submerged in an ice-water bath to cool to around 20°C to allow settling and to prevent further hydrolysis. The hydrolysate obtained was separated from the residual lignocellulosic fiber by filtration through a 100 mesh sieve filter and centrifuged at 12 000 rpm for 15 min. The supernatant, referred as sago hampas hydrolysate (SHH), was harvested and analyzed for reducing sugars and glucose content (analytical procedures). The pellet (lignocellulosic fiber) was oven dried before being observed for its physical structure by Scanning Electron Microscope (SEM). The same procedure of enzymatic treatment was repeated for 7%, 9%, 12%, and 15% of sago hampas suspension, respectively. Three replicates were done on each concentration of sago hampas. 

For hydrolysis yield (%) in this study, it was calculated as follows:

(1) $$\begin{array}{c} \frac{\text{Glucose}\;\text{produced}\;\text{from}\;\text{starch}\;\text{of}\;\text{sago}\;\text{hampas}\;\left(\text{g}\right)}{\text{Dry}\;\text{sago}\;\text{hampas}\;\left(\text{g}\right)}\times 100. \end{array}$$

### 2.4. Increasing Glucose Concentration

**Figure 1:**
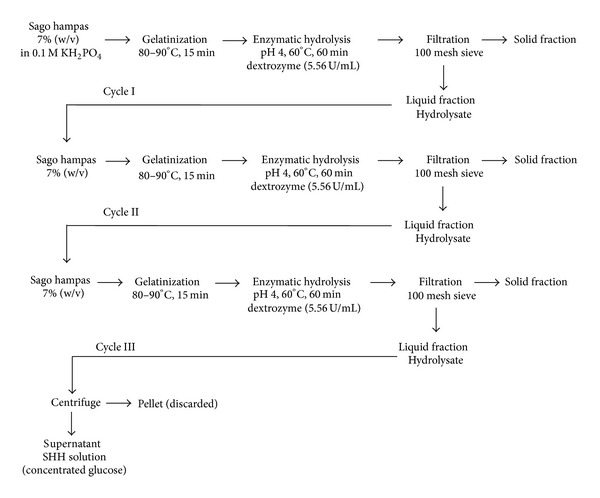
Schematic diagram of increasing glucose concentration from sago hampas by three cycles of enzymatic hydrolysis.

In order to achieve a sufficient amount of glucose in SHH, three cycles of enzymatic hydrolysis process were conducted (Figure 1). Initially the same procedure of enzymatic hydrolysis process was conducted (refer to the above section), and this stage was known as cycle I. Once the hydrolysis was completed, the hydrolysate was filtered. The liquid portion was reused for cycle II whereas the solid part was oven dried for further pretreatment [5]. Before hydrolysis was carried out for cycle II, the volume of hydrolysate obtained during cycle I was measured in order to ensure the amount of new dried sago hampas loaded was based on the basis of 7% (w/v). Usually the volume of hydrolysates lost was about 20% at the end of cycle III enzymatic hydrolysis process, due to evaporation (during saccharification stage, temperature was fixed at 60°C) and filtration (during liquid-solid separation after hydrolysis process was completed). The procedure of enzymatic hydrolysis process was repeated for the second and third cycles. The hydrolysate was centrifuged once during the completion of the third cycle of hydrolysis. Here higher glucose concentration (g/L) was expected in SHH so that it was ready for use as substrate for ethanol fermentation.

### 2.5. Analytical Procedures

Moisture content was determined by drying at 105°C to constant weight [19]. The analysis of cellulose, hemicellulose, and lignin of sago hampas was determined according to Goering and Van Soest (1970) [20]. Starch content was estimated by Iodine Starch colorimetric method [21]. Glucose and oligosaccharides were analyzed by High Pressure Liquid Chromatography (HPLC) system (Shimadzu, Kyoto, Japan), equipped with Shimadzu Liquid Chromatograph (LC-20AT) and Shimadzu Refractive Index Detector (RID-10A). The column used was Aminex Fermentation Monitoring Column 150 mm × 7.8 mm, whereas 5 mM H_2_SO_4_ was used as a mobile phase with a flow rate of 0.8 mL/min at 60°C.

### 2.6. Scanning Electron Microscopy (SEM) of Physically and Enzymatically Treated Sago Hampas

Physically and enzymatically treated sago hampas samples were prepared for SEM observation by sprinkling it on double-sided adhesive tape attached to a circular specimen stub coated with platinum. The microstructure of the samples was viewed via JEOL, JSM-6390LA Scanning Electron Microscope (SEM) for observation of starch granules and changes occurring on cluster of sago hampas. 

### 2.7. Fermentation of Enzymatic Hydrolysate

The hydrolysate of sago hampas enzymatic treatment was fermented to observe the ability to produce ethanol in batch system utilizing commercial baker's yeast, *S. cerevisiae*. The hydrolysate was supplemented with 3 g/L yeast extract, 1 g/L peptone, 1.4 g/L (NH_4_)_2_SO_4_, 2 g/L KH_2_PO_4_, and 0.3 g/L MgSO_4_·7H_2_O. The glucose concentration in SHH was set at 80 g/L and commercial glucose was used as control. The yeast (Mauripan Baking Industry) which was cultured on potato dextrose agar and yeast peptone glucose agar was transferred into 100 mL inoculum media containing 20 g/L glucose and 5 g/L yeast extract. The inoculum was incubated for 9 h at 30°C before being centrifuged at 8000 rpm for 5 mins to obtain the cell pellet which then was ready to be added into fermentation media. The fermentation was carried out at 30°C, 100 rpm, and initial pH 5.5-5.6. The samples withdrawn were centrifuged at 10,000 rpm for 10 min at 4°C and the cell free supernatant was used for the determination of ethanol produced and glucose consumed. Ethanol concentration in the fermentation broth was determined using the same HPLC configuration as for glucose. The ethanol yield (*Y*_*p*/*s*_) was calculated as the actual ethanol produced and expressed as g ethanol per g glucose utilized (g/g). The percentage of conversion efficiency based on theoretical yield was calculated by *Y*_*p*/*s*_/0.51 × 100. The volumetric ethanol productivity was calculated by actual ethanol concentration produced (g/L) per fermentation time (h) giving the highest ethanol concentration.

## 3. Results and Discussion

**Figure 2:**
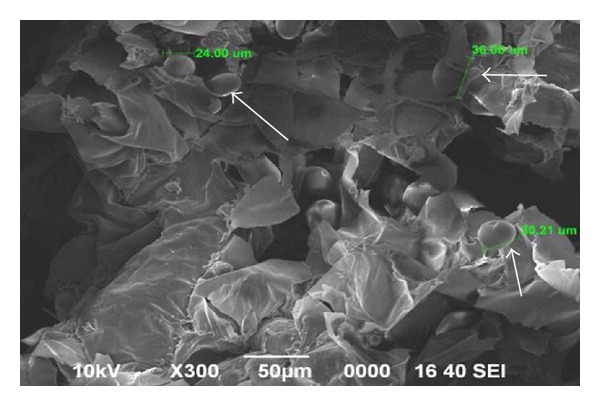
Scanning electron microscope photograph of untreated sago hampas. Starch granules (white arrow) were trapped within the sago hampas.

The compositions of dried sago hampas is shown in Table 1. Sago hampas, the solid waste produced after starch extraction, contains a significant proportion of starch granule material and fiber (Figure 2). According to Chew and Shim (1993), microscopic examination revealed a large number of starch granules to be trapped within the lignocellulosic matrix of sago hampas [22]. The sago starch granules were either pear or cigar shaped and had a generally smooth outer surface with some shallow indentations whereas the size distribution was in a narrow range of 10–50 *μ*m with a mean size of 32 *μ*m [23].

All values except starch are comparable to those reported previously [24, 25]. In this study, low amount of starch in sago hampas was observed due to the quality of the extraction process practiced by sago mill as it greatly depended on the sophistication of the methods employed [26]. Moreover, sago industry is still under development, and therefore every year the factory owners will try to improve their processing to minimize the starch content in sago hampas. According to one owner, most of the factory that achieves food grade for their starch production will have more starch in the sago hampas compared to the factory that produces industrial grade starch. This is due to the reduced recycling process which was carried out during the starch extraction stage, to ensure the starch whiteness.

Initially, the study was carried out to identify the effects of sago hampas concentration, (w/v: 5%, 7%, 9%, 12%, and 15%) on enzymatic hydrolysis using dextrozyme (5.56 U/mL). Before saccharification process was carried out, the sago hampas suspension underwent gelatinization stage for at least 15 mins. Gelatinization possibly will disrupt the sago starch granules, destroying the crystallites, and the granules will be susceptible to enzyme attack [27]. The addition of dextrozyme to the heated gelatinized sago hampas suspensions resulted in a more runny solution after 15 mins of reaction, especially for 5% and 7% suspension. However, the suspension of sago hampas for 9%, 12%, and 15% was very viscous, thus low yield of glucose was observed at the end of hydrolysis. As shown in Table 2, analysis of the hydrolysates upon completing cycle I, obtained from different sago hampas suspension, revealed that the glucose levels obtained increased with increasing substrate load from 5% to 9% only. However, when enzymatic hydrolysis was carried out at 12 of substrate load, the glucose concentration starts to decline. The same phenomena was also observed at 15% sago hampas suspension. The conversion yield, however, shows some decline starting at 9% substrate concentration which reveals that the enzymatic reaction at high insoluble solid consistency leads to increased viscosity, higher energy requirement for mixing, and shear inactivation of enzymes, as well as poor heat transfer due to rheological properties of dense fibrous suspension [28].

**Figure 3:**
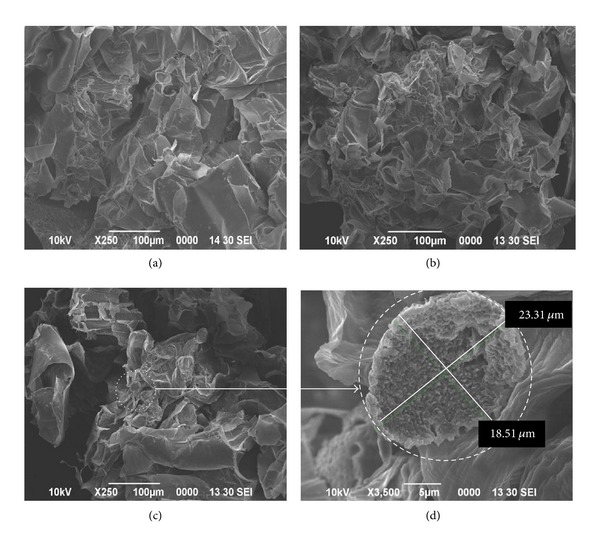
Scanning electron microscope photograph of sago hampas with different suspension (w/v) of sago hampas after enzymatic treatment. No starch residue was trapped within sago hampas after enzymatic hydrolysis on 5% suspension (a) and on 7% suspension (b). Incomplete hydrolysis of starch was observed in 9% sago hampas suspension (c), and enlarged image of starch in 9% sago hampas suspension (d).

From Figures 3(a) and 3(b), SEM photographs show no starch present in 5% and 7% of treated sago hampas. This suggested that the enzyme had hydrolyzed all the trapped starch. Moreover, the sago hampas slurry did not turn blue after the addition of iodine solution, indicating the hydrolysis of most to all of the starch. However, in 9% treated sago hampas, some starch still existed as observed under SEM, Figures 3(c) and 3(d). A reduction of water content is expected to complicate the processing and will lead to an increasing viscosity of the reaction mixture as well as an increasing melting temperature of starch [29]. This indicated that some starch was not melted during gelatinization process at 90°C for 15 mins, thus incomplete saccharification process for 9% suspension was encountered.

Macromolecules within native sago starch were not as susceptible to hydrolysis as in gelatinized sago starch. The debranching enzyme, namely, pullulanase, acts on the released, soluble oligosaccharides rather than on the granule material [30].

Starch in sago hampas was bounded by the structural and physical properties of lignocellulosic materials, thus influencing the accessibility of enzymes to the substrate. As stated by Andersson et al. [31], cell walls in plant cell consist of microstructural cellulose embedded in a polysaccharide and protein matrix, surrounded by an outer layer of pectic material. Thus, starch granules inside this complex polymer matrix are difficult to liberate. The increase of sago hampas concentration up to 9% (w/v) or more will cause the enzymatic hydrolysis process to be difficult, thus leading to lower hydrolysis yield. Sugar concentration after hydrolysis of lignocellulosic materials is often low due to challenges in feeding solids concentrations higher than 10% by weight and end product inhibition of cellulase enzymes by the sugars released [32].

Some other factors might contribute to lower hydrolysis yield were the inhibition of polyphenols in the sago waste [33]; product inhibition [34] and the decreased affinity of dextrozyme towards the substrate [35]. Application of enzyme mixture such as cellulase and pectinase can actually increase the efficiency of starch recovery from starchy agricultural waste, thus higher reducing sugar can be converted [36]. Another approach introduced to achieve higher concentrations of sugar was through concentration step utilizing vacuum evaporation [37]. However, the supplementation with cellulase or pectinase would add to the already substantial cost for enzyme in the bioconversion process. The removal of sugars by ultrafiltration or evaporation will also contribute to a high-cost process, thus restricts its large-scale application. Thus the strategy of three-cycle enzymatic hydrolysis process of sago hampas treated as raw material for glucose production and utilizing dextrozyme alone was deemed sufficient to obtain high glucose concentration.

Table 3 represents the glucose concentration (g/L) and hydrolysis yield (%) for three-cycle enzymatic hydrolysis process. For each cycle, 7% (w/v) of sago hampas suspension was prepared, thus total substrate load was accounted to be 21% (w/v). In the observation, conducting the hydrolysis process was much easier for cycle II and III because the property of substrate solubilization during enzymatic hydrolysis was much better compared to handling hydrolysis in cycle I due to excess enzyme in suspension, enabling agitation to be carried out properly thus leading to better heat and mass transfer distribution. Moreover, feeding substrate batch by batch into the hydrolysis system is important for the interaction between substrate and enzymes because water (buffer) content in the suspension is also crucial for enzyme transport mechanisms throughout hydrolysis as well as mass transfer of intermediates and end products [38]. It was also observed that recycled hydrolysate led to the achievement of higher glucose concentration in the subsequent cycles due to total glucose accumulated that was based on glucose produced at the previous cycle plus glucose produced in the current cycle. The concentration of glucose after cycle I of enzymatic hydrolysis was 27.79 g/L, with 35.73% of hydrolysis yield. When SHH solution from cycle I was used for subsequent enzymatic saccharification, 73.00 g/L glucose was produced at the end of cycle II, and better hydrolysis yield (44.32%) was achieved. The improvement of hydrolysis yield (52.72%) was again observed at the end of cycle III, showing indications of glucose production as high as 138.45 g/L.

An improvement of overall glucose production, hydrolysis yield and hydrolysis rate was observed after conducting three-cycles enzymatic hydrolysis process for sago hampas. Indeed, the 138.45 g/L glucose seen in the hydrolysate after the third cycle of hydrolysis represents some 52.72% (w/w) of the total mass of sago hampas, and close to the 58% (w/w) starch composition [5], suggesting a high degree of saccharification. The existance of glucose in the previous hydrolysate shows no interruptions or even inhibition in the subsequent enzymatic hydrolysis process. Each cycle shows that 30 minutes was enough (data not shown) for the saccharification process as no increment of glucose concentration was observed when the time was prolonged to one hour. The hydrolysis process shows better conversion yield at the early stage of the saccharification process due to preferential hydrolysis of the amorphous region, and the rate decreased as the enzyme encountered the more recalcitrant crystalline region [39]. Further analysis using HPLC revealed that instead of glucose as the main component, dextrin, maltose, and maltotriose were also exists in SHH at all stages of hydrolysis. The same components of the reducing sugars from hydrolyzed sago pith substrate were reported before [8]. According to the analysis, glucose content of total reducing sugars found in SHH was about 85%–90% (w/v). Thus, higher composition of glucose in SHH creates an extra advantage as it can be used as carbon sole for ethanol fermentation. 

 It was experimentally demonstrated that high glucose concentration can be obtained when hydrolysate was used for subsequent hydrolysis process in which more substrate loads can be fed into the hydrolysis system—thus can avoid evaporation or reduced water to be evaporated if higher glucose concentration was needed for ethanol production. However, some drawbacks such as more brownish color of hydrolysate are observed once the three-cycle hydrolysis was completed and the losses of hydrolysate volume up to 20% at the end of the process. Future study on color removal such as that by activated charcoal and proper close system reactor used for conducting hydrolysis might minimize those drawbacks.

**Figure 4:**
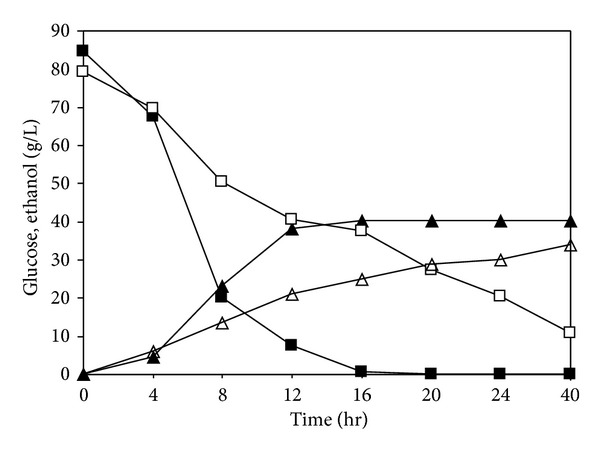
The profiles of glucose and ethanol concentrations during ethanol fermentation utilizing sago hampas hydrolysate by baker yeast at 30°C (■-glucose of SHH, ▲-ethanol from SHH, □-commercial glucose, and ∆-ethanol from commercial glucose).

Figure 4 indicated the preliminary study on the ability of SHH as a substrate for ethanol production via batch fermentation system utilizing commercial baker's yeast. The fermentation process produced 40.30 g/L ethanol from 84.75 g/L of glucose in SHH after 16 hours. This is equivalent to 93.29% of conversion yield based on total glucose existing in fermentation media. For comparison, 92.00% of conversion yield was observed when commercial glucose was used as substrate. The ethanol volumetric productivity of 2.52 g/Lh was obtained in fermentation media containing glucose of SHH, whereas it was1.50 g/Lh when utilizing commercial glucose as carbon source. In ethanol fermentation using *Zymomonas mobilis* from simultaneously saccharified sago starch, 2.91 g/Lh of ethanol volumetric productivity was obtained [40]. On the other hand, Bandaru et al. (2006) reported that 3.21 g/Lh of ethanol volumetric productivity was achieved in optimized fermentation conditions using sago starch by coimmobilized amyloglucosidase with *Zymomonas mobilis* [17]. As an overall, the glucose obtained from enzymatic hydrolysis of trapped starch in sago hampas has shown the same capability with glucose obtained from primary sago starch when used as substrate by commercial baker's yeast for bioethanol production. 

## 4. Conclusion

The properties of sago hampas were affected by its structure and the characteristics of starch and lignocellulose compound, thus enzymatic hydrolysis process was difficult to be carried out when higher substrate load was used. The 7% (w/v) of sago hampas suspension was suitable for enzymatic hydrolysis using dextrozymes with respect to glucose production and conversion yield. However, to increase glucose concentration (g/L), the strategy of conducting three cycles of sago hampas enzymatic hydrolysis was seen to be practical. High proportion of glucose compared to other constituents in hydrolysate is another advantage as it can serve as a suitable substrate by most of the microorganism for production of value-added products. The ability of this glucose for bioethanol production also proved sago hampas was found to serve as an excellent raw material as well as representing an alternative and readily manageable option.

## Figures and Tables

**Table 1:** Compositional analysis of sago hampas.

Composition	% (dry basis)
Starch	30–45
Moisture	5–7
Ash	3-4
Protein	1
Fiber	30–35
Fat	ND
pH	4.6-4.7

ND: not detected.

**Table 2:** Glucose production from different concentration of sago hampas suspension after being treated with dextrozyme (5.56 U/mL) upon completing cycle I.

Sugars	Sago hampas suspension (w/v)				
	5%	7%	9%	12%	15%
Reducing sugar (g/L)	17.23 ± 0.85	31.12 ± 1.22	35.26 ± 1.45	34.22 ± 0.89	28.76 ± 2.20
Glucose (g/L)	15.30 ± 1.20	27.79 ± 1.85	31.74 ± 1.55	30.80 ± 1.35	25.88 ± 1.95
Conversion yield (%)	30.60 ± 2.4	39.71 ± 2.6	35.27 ± 1.71	25.66 ± 1.13	17.25 ± 1.30

**Table 3:** Glucose production and hydrolysis yield of three-cycle enzymatic hydrolysis.

Hydrolysis scheme	Glucose (g/L)	Hydrolysis yield (%)^a^	Hydrolysis rate (g/L·min)^b^
Cycle I	27.79 ± 1.85	35.73	0.93
Cycle II	73.00 ± 3.50	44.32	1.53
Cycle III	138.40 ± 2.11	52.72	2.17

Notes: a = {glucose(g)/sago  hampas(g)} × 100%; b = {[glucose]_30min⁡_ − [glucose]_0min⁡_}/30 min⁡.
